# Fine mapping of *Rcr1* and analyses of its effect on transcriptome patterns during infection by *Plasmodiophora brassicae*

**DOI:** 10.1186/1471-2164-15-1166

**Published:** 2014-12-23

**Authors:** Mingguang Chu, Tao Song, Kevin C Falk, Xingguo Zhang, Xunjia Liu, Adrian Chang, Rachid Lahlali, Linda McGregor, Bruce D Gossen, Fengqun Yu, Gary Peng

**Affiliations:** Department of Agriculture and Agri-Food Canada (AAFC), Saskatoon Research Centre, 107 Science Place, Saskatoon, Saskatchewan S7N 0X2 Canada

**Keywords:** Clubroot, *Plasmodiophora brassicae*, Genetic mapping, Marker-assisted selection, Next-generation sequencing, RNA-seq, Gene ontology, Transcription factors

## Abstract

**Background:**

The protist *Plasmodiophora brassicae* is a biotrophic soil-borne pathogen that causes clubroot on Brassica crops worldwide. Clubroot disease is a serious threat to the 8 M ha of canola (*Brassica napus*) grown annually in western Canada. While host resistance is the key to clubroot management, sources of resistance are limited.

**Results:**

To identify new sources of clubroot resistance (CR), we fine mapped a CR gene (*Rcr1*) from *B. rapa* ssp. c*hinensis* to the region between 24.26 Mb and 24.50 Mb on the linkage group A03, with several closely linked markers identified. Transcriptome analysis was conducted using RNA sequencing on a segregating F_1_ population inoculated with *P. brassicae*, with 2,212 differentially expressed genes (DEGs) identified between plants carrying and not carrying *Rcr1*. Functional annotation of these DEGs showed that several defense-related biological processes, including signaling and metabolism of jasmonate and ethylene, defensive deposition of callose and biosynthesis of indole-containing compounds, were up-regulated significantly in plants carrying *Rcr1* while genes involved in salicylic acid metabolic and signaling pathways were generally not elevated. Several DEGs involved in metabolism potentially related to clubroot symptom development, including auxin biosynthesis and cell growth/development, showed significantly lower expression in plants carrying *Rcr1*.

**Conclusion:**

The CR gene *Rcr1* and closely linked markers will be highly useful for breeding new resistant canola cultivars. The identification of DEGs between inoculated plants carrying and not carrying *Rcr1* is an important step towards understanding of specific metabolic/signaling pathways in clubroot resistance mediated by *Rcr1*. This information may help judicious use of CR genes with complementary resistance mechanisms for durable clubroot resistance.

**Electronic supplementary material:**

The online version of this article (doi:10.1186/1471-2164-15-1166) contains supplementary material, which is available to authorized users.

## Background

Clubroot, caused by the biotrophic protist *Plasmodiophora brassicae* Woronin, is one of the most serious diseases of Brassica crops worldwide [1]. In western Canada, clubroot disease has become a major threat to the production of canola (*Brassica napus* L) [2], where more than 8 M ha of canola crops are grown annually [3]. The pathogen is able to survive for up to 20 years in soil [4] and many conventional disease-management measures, including cultural techniques and application of fungicides, are not effective [3, 5, 6]. Genetic resistance is the most effective and economical approach to clubroot management on canola. European fodder turnips (*Brassica rapa* L. ssp. *rapifera*) are the major source of clubroot-resistance (CR) genes, which have been introduced into other Brassica crops including oilseed rape (*B. napus*), rutabaga (*B. napus* L. ssp. *napobrassica*) and Chinese cabbage (*B. rapa* L. ssp. c*hinensis*) [7–11].

Since 2009, several resistant (R) canola cultivars have been released in Canada, and all of them carry a single dominant CR gene. The source and genetic information are not revealed for these CR genes [12]. The durability of these clubroot R cultivars remains unknown in western Canada, but resistance conferred by a single gene is generally not durable. Breakdown of clubroot resistance has been reported on Chinese cabbage [13] and oilseed rape [14, 15]. A resistant canola cultivar showed substantially increased clubroot severity after being exposed to pathotype 3 of *P. brassicae* after only two cycles under controlled conditions [16]. Rotation or pyramiding of CR genes with different mechanisms of resistance may be used to increase the durability of clubroot resistance if a diverse group of CR genes can be identified and their resistance mechanisms characterized. Our prior work evaluated 955 *Brassica* accessions and identified a range of CR candidates from *B. rapa, B. nigra* and *B. oleracea*[17].

Most of the known CR genes have been identified from *B. rapa*, with eight loci reported previously: *Crr1, Crr2, Crr3, Crr4, CRa, CRb, CRc* and *CRk*[18–22]. *CRa* and *Crr1* have been isolated recently [23, 24]. Another CR gene, *RPB1*, was identified from *Arabidopsis thaliana* ecotype Tsu-0 [25], but there has been no further report on its orthlogs in other *Arabidopsis* ecotypes. A new CR gene (*Rpb1*) was identified recently from the cv. Flower Nabana (FN) of pak choy (*B. rapa* ssp. *chinensis*) via rough mapping [26]. *Rpb1* is identical to *Rcr1*described in this paper, and the name change was to avoid potential confusion with the RPB1 from *Arabidopsis*. There has been little information on molecular mechanisms associated with any of the CR genes reported. In *A. thaliana*, host metabolism was altered by *P. brassicae* infection; transcriptome studies based on microarray analysis showed that genes encoding enzymes involved in carbohydrate metabolism were upregulated in root tissues of the susceptible (S) Col-0 ecotype [27, 28], but not in moderately resistant (MR) ecotypes which appeared to reduce or delay pathogen-triggered metabolic diversion and cell enlargement or proliferation in the host [29]. Reduced trehalose and arginine metabolism were also reported with the partially resistant *A. thaliana* ecotype Bur-0 when compared with that in a susceptible ecotype [30, 31]. Secondary metabolism, including flavonoids, may also contribute to formation of characteristic club symptoms in Arabidopsis, and inhibition of oxoglutaric acid-dependent dioxygenases reduced club development [32]. Treatment with the phytohormone salicylic acid or biofungicides reduced clubroot development on *A. thaliana* and *B. napus* via activation of several defense-related pathways in the hosts [33–36]. However, there is no information on molecular mechanisms of clubroot resistance in *Brassica* species based on transcriptome analysis. RNA sequencing (RNA-seq) has been employed recently to elucidate resistance mechanisms involved in plant-pathogen interactions including *Sclerotinia homoeocarpea*-creeping bentgrass [37] and *Phytophthora infestans*-potato tuber [38].

In the present study, we intended to: 1) identify and characterize the CR gene from a highly resistant pak choy cultivar using genetic mapping; 2) develop molecular markers closely linked to this CR gene to facilitate marker-assisted selection (MAS) at the young seedling stage; and 3) analyze the global transcriptome profile associated with the CR gene based on RNA-seq. We examined differential gene expression between R and S F_1_ plants, and the result provided important insights into the molecular mechanisms of clubroot resistance. This work also sets the first step toward the development of canola germplasm using CR genes with potentially different modes of action against clubroot.

## Results

### The clubroot resistance in cv. FN is associated with a single dominant allele

**Figure 1:**
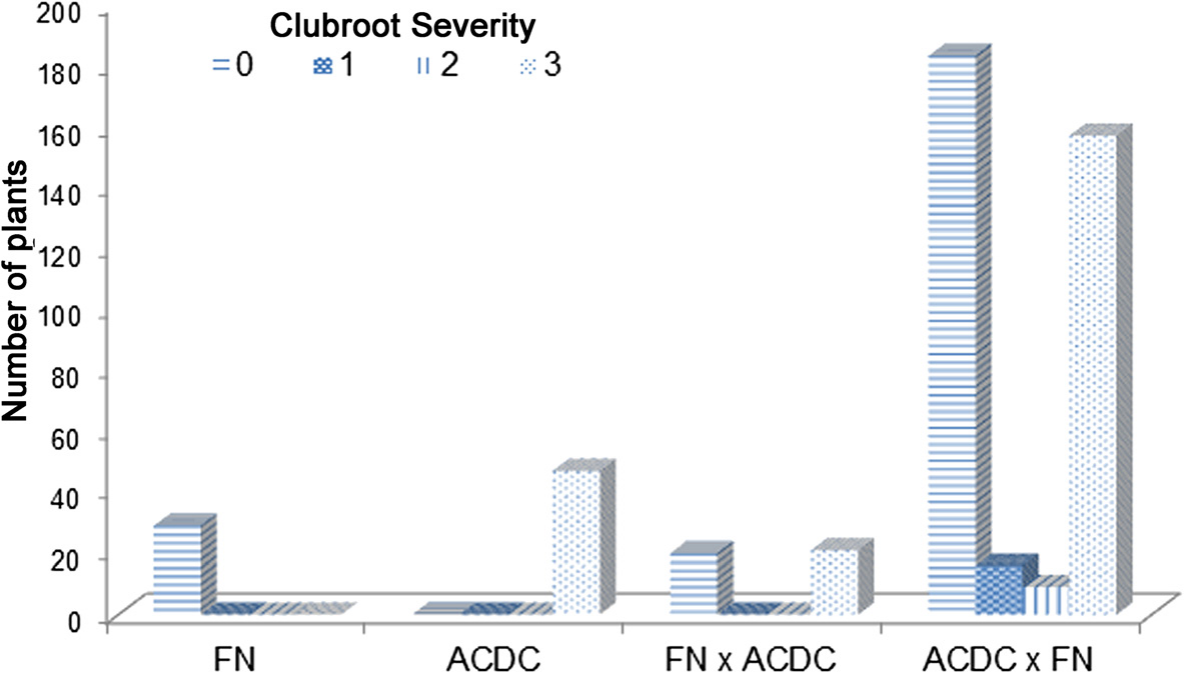
**Segregation in clubroot resistance for parents (FN and ACDC) and F**_**1**_ **populations derived from reciprocal crosses (FN × ACDC and ACDC × FN, respectively).** These F_1_ plants were not part of the F_1_ population (1,587 plants) used later for fine mapping of CR genes.

All of the FN plants were resistant to pathotype 3 of *P. brassicae*, showing no clubroot symptom at 5 weeks after inoculation, whereas all of the ACDC plants were susceptible (Figure 1). Analyses of the F_1_ populations from reciprocal crosses showed a segregation pattern that would fit a 1:1 ratio between R and S plants (*X*^2^ = 2.98, *P* = 0.084), indicating that the resistance in FN is associated with a single dominant nuclear gene. This gene was designated as *Rcr1* (previously *Rpb1*)*.* Also, the pattern of clubroot disease response in parental and F_1_ populations (Figure 1) indicated that the *Rcr1* locus was likely heterozygous in the cv. FN.

### Fine mapping of the gene *Rcr1*and development of molecular markers

**Figure 2:**
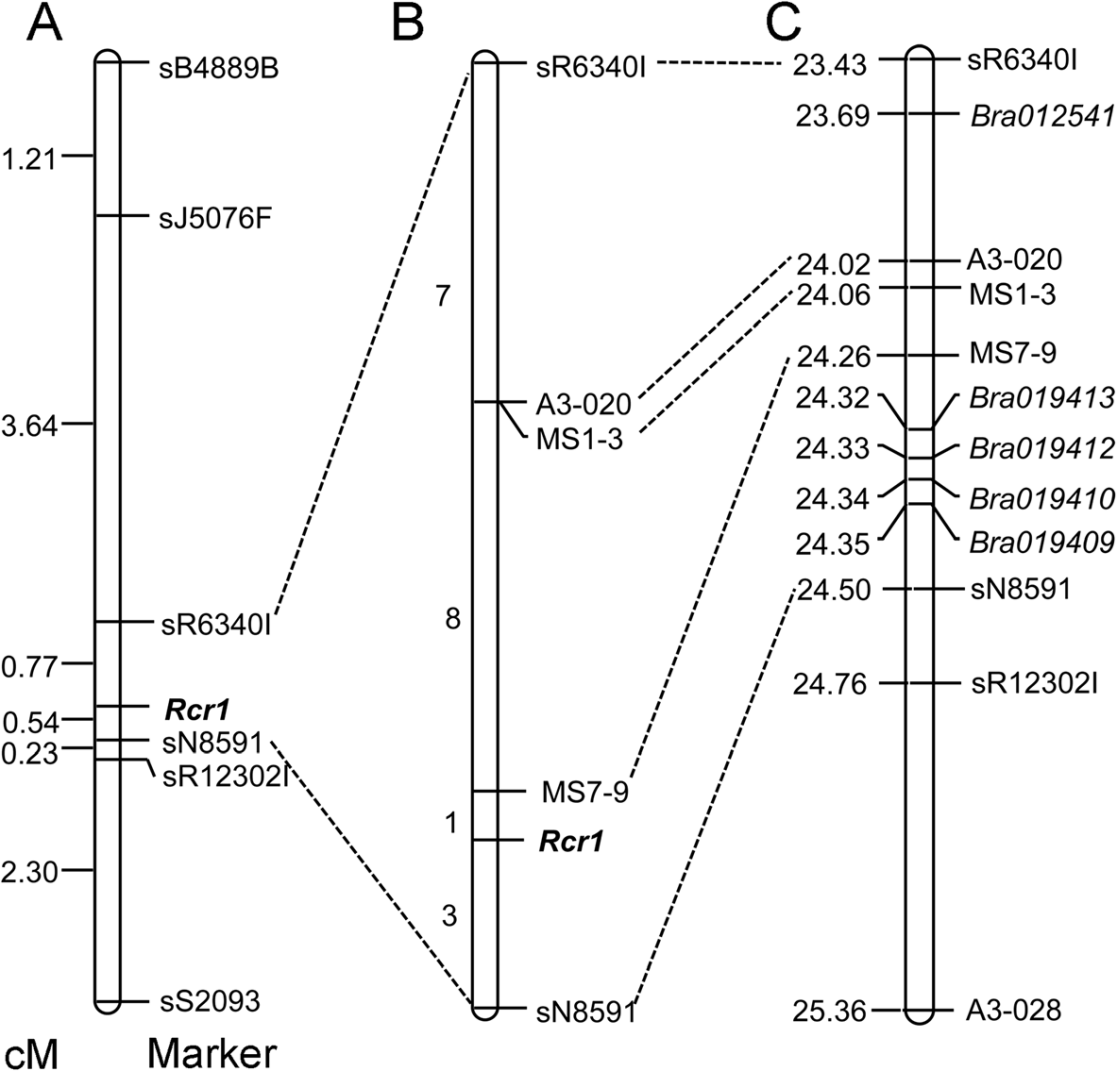
**Linkage maps of the regions in which the** ***Rcr1*** **gene is located. Broken lines drawn regions defined by different molecular markers on** ***B. rapa*** **linkage group A03. A)** Rough mapping of *Rcr1* based on a small F_1_ population (300 plants) derived from ACDC × FN. The genetic distance is shown on the left. **B)** Fine mapping of *Rcr1* based on 1,587 F_1_ plants. **C)** Physical locations in Mb (left) of the molecular markers and TIR-NBS-LRR genes in the region flanked by the markers sN8591 and sR6340I.

*Rcr1* was roughly mapped to a range of 1.31 cM in the *B. rapa* linkage group A03 flanked by the markers sN8591 and sR6340I (Figure 2A), and fine mapping was based on testing additional 1,587 F_1_ plants using pathotype 3 of *P. brassicae* and on analysis using these flanking markers (Figure 2B). The flanked segment is homologous to the region between 23.43 Mb and 24.50 Mb on the A03 (*B. rapa* reference genome sequence, Chromosome v1.2), with 158 genes annotated (http://brassicadb.org/brad) and five of them (*Bra012541, Bra019409*, *Bra019410*, *Bra019412* and *Bra019413*) identified as encoding toll interleukin-1 receptor (TIR)- nucleotide-binding site (NBS)-leucine-rich repeat (LRR) class of proteins (Figure 2C). *Bra012541* is located close to 23.69 Mb and the rest were in a cluster located between 24.32 Mb and 24.35 Mb.

**Figure 3:**
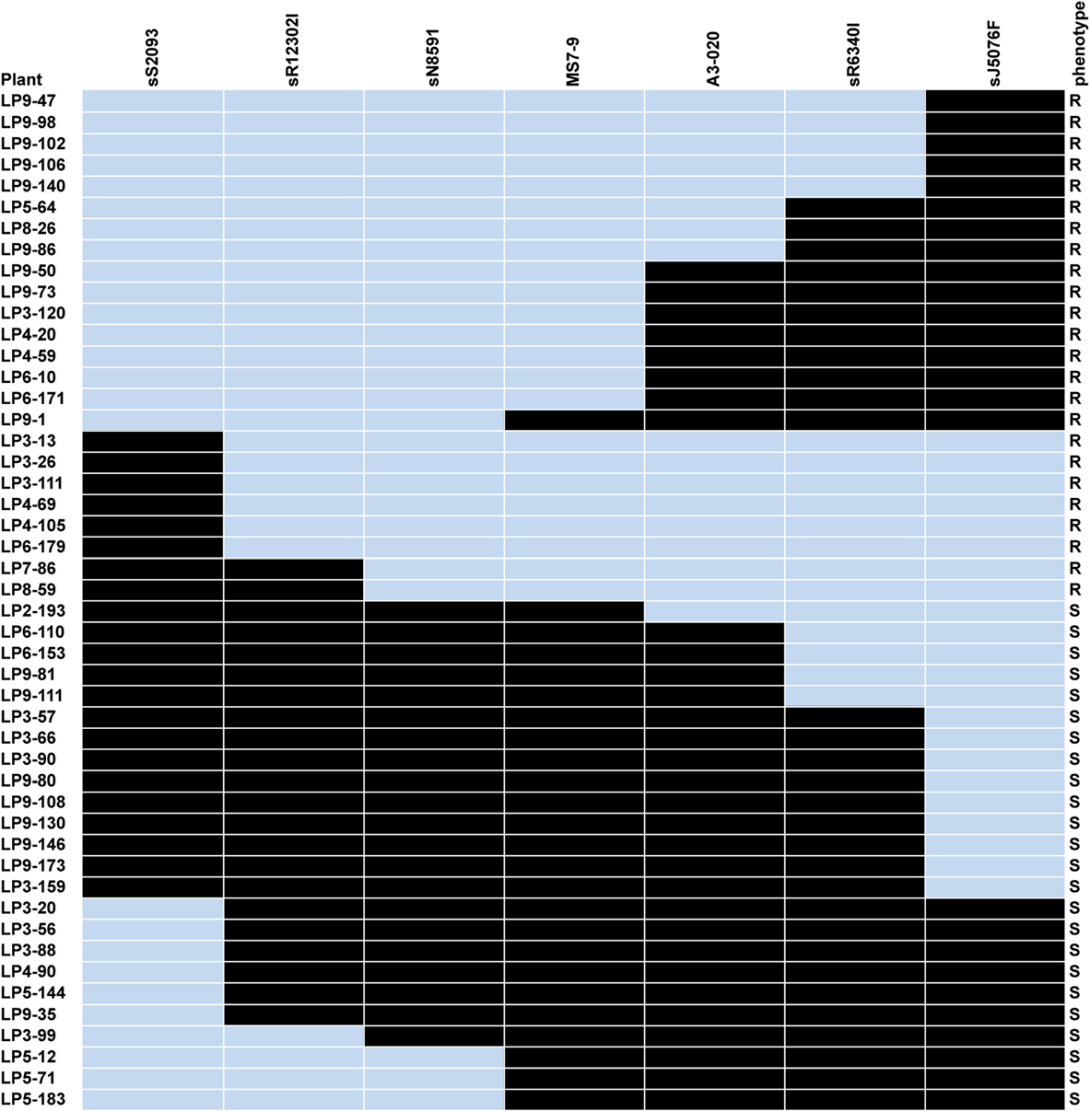
**Genotypes and phenotypes of recombinants selected from the mapping population inoculated with pathotype 3 of** ***Plasmodiophora brassicae*** **.** Line identifications and phenotypes (R for resistant, S for susceptible) are denoted on the left and right, respectively, with marker names at the top. Resistance alleles are denoted in light grey and susceptible alleles in black. The two markers in a grey shadow flank the narrowest interval containing the *Rcr1* gene.

**Figure 4:**
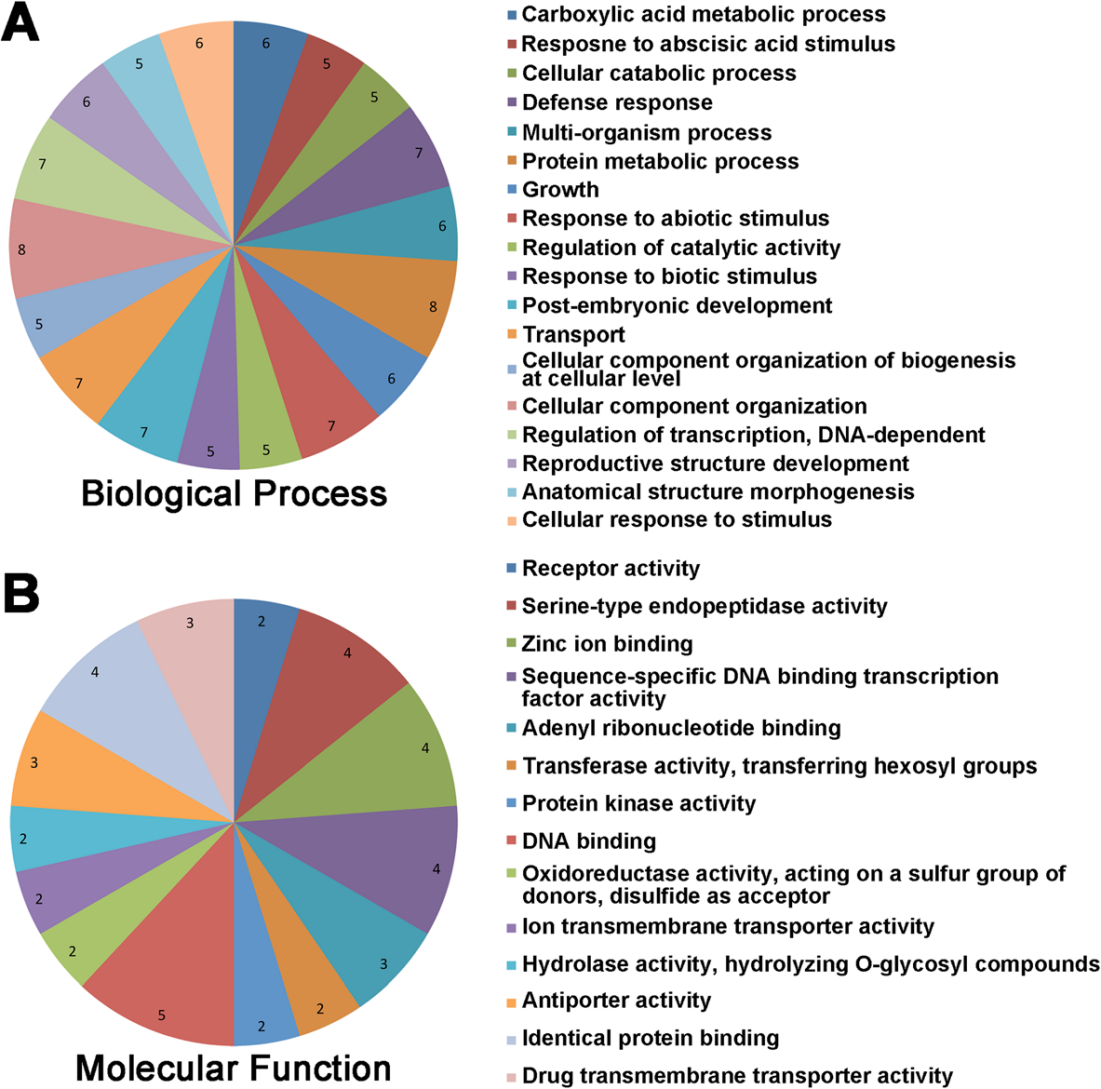
**Gene ontology (GO) annotations of genes residing in the region flanked by the markers sN8591 and sR6340I in fine mapping:** GO terms in the category of A) Biological Process and **B)** Molecular Functions. The value labeled in the pie chart of both **A)** and **B)** are the number of genes annotated with the corresponding GO term.

A total of 19 recombinants were identified via comparison of marker and phenotype data over the 1,587 F_1_ plants (Figure 3), with 3 falling between sN8591 and *Rcr1* and 16 between *Rcr1*and sR6340I. A CAPS marker (A3-020), homologous to *Bra038794* at 24.02 Mb, was developed for further analysis of the 19 recombinants, and showed an approximate distance of 0.57 cM from *Rcr1*, which was closer to the CR gene than sR6340I. The interval flanked by sN8591 and A3-020 was estimated at 0.76 cM, consisting of approximately 480 Kb with 67 genes annotated (Additional file 1: Table S2). The CAPS marker MS7-9 (5′-AGAGGCTTTCTCCATCAA-3′, 5′-GACATAAGAATCCCACAA-3′) was identified slightly later and appeared even closer to *Rcr1* than A3-020 (Figure 2B). Based on the rate of recombination, the genetic distance of *Rcr1* was estimated at 0.19 cM from sN8591 and 0.06 cM from MS7-9, respectively. The cluster of four TIR-NBS-LRR genes and one defense-related gene (*Bra019401*, ccr4-associated factor 1b) are located also within this interval. The gene ontology (GO) terms for these genes are in Table 1 and Figure 4.

**Table 1:** **The defense-related genes annotated within the fine mapped region flanked by the markers sN8591 and A3-020 in the** ***Brassicae rapa*** **linkage group A03 and their associated gene ontology (GO) terms**

Seq. ID	Seq. description	GO term
*Bra019401*	ccr4-associated factor 1b	P: Intracellular signal transduction; F: Ribonuclease activity; P: Ethylene biosynthetic process; P: RNA modification; P: Abscisic acid mediated signaling pathway; C: nucleus; P: Ethylene mediated signaling pathway; F: Nucleic acid binding; P: Defense response, incompatible interaction; P: MAPK cascade; P: Respiratory burst involved in defense response; P: defense response to bacterium; C: Intracellular; P: Nuclear-transcribed mRNA poly(A) tail shortening; P: Vegetative to reproductive phase transition of meristem; P: Response to chitin; F: 3′-5′ exonuclease activity; P: Response to biotic stimulus; P: Response to wounding
*Bra019407*	autophagy-related protein 8a	F: Receptor activity; F: Microtubule binding; P: Para-aminobenzoic acid metabolic process; C: Autophagic vacuole; F: APG8-specific protease activity; P: Defense response to fungus; P: Heat acclimation; F: APG8 activating enzyme activity; C: Vacuolar lumen; F: Atg8 ligase activity; P: Autophagy
*Bra019409*	tir-nbs-lrr class resistance protein	P: Defense response to bacterium; F: Adenyl ribonucleotide binding
*Bra019410*	disease resistance protein	P: Defense response to bacterium; F: Nucleotide binding
*Bra019412*	tir-nbs-lrr class resistance protein	F: Nucleoside-triphosphatase activity; P: Defense response; F: ADP binding; P: Signal transduction; C: Intracellular
*Bra019413*	tir-nbs-lrr class resistance protein	C: Golgi membrane; C: Endoplasmic reticulum membrane; F: Binding; P: Defense response to fungus, incompatible interaction; C: Plasma membrane; P: Response to oomycetes
*Bra038776*	cysteine-rich receptor-like protein kinase 29	C: Vacuole; P: Response to chitin; C: Plasma membrane; P: Respiratory burst involved in defense response; P: Protein phosphorylation; F: ATP binding; P: Response to abscisic acid stimulus; F: Protein serine/threonine kinase activity

### Validation of selected markers for detection of *Rcr1*in backcross populations

On the BC_1_*B. napus* population, sN8591 detected *Rcr1* in 99.8% of the resistant (R) and 0.2% of susceptible (S) plants, while sR6340I detected the CR gene in 95.9% of R and 4.1% of S plants, respectively (Table 2). On *B. rapa*, however, the accuracy was slightly poorer for both sN8591 (96.5% of R, 3.5% of S) and sR6340I (92.7% of R, 7.3% of S). The accuracy was much poorer for the markers sB4889B and sS2093 on both *B. napus* and *B. rapa*, with erroneous identification of *Rcr1* at >7.3%.

**Table 2:** **Validation of flanking markers for detecting the** ***Rcr1*** **gene (%) in clubroot resistant BC**_**1**_ **progeny**

	***B. napus*** ^***a***^		***B. rapa*** ^***a***^	
Molecular markers	Resistant ^***b***^	Susceptible ^***b***^	Resistant	Susceptible
sN8591	99.8%	0.2%	96.5%	3.5%
sR6340I	95.9%	4.1%	92.7%	7.3%
sB4889B	79.4%	20.6%	87.8%	12.2%
sS2093	92.7%	7.3%	80.2%	19.8%

^*a*^The BC_1_ populations were derived from crosses of a DH line of *B. napus* (SV11-17667) and *B. rapa* (BH11-17938), respectively, with cv. FN. Each BC_1_ population use for the experiment consisted of 176 plants.

^*b*^“Resistance” and “Susceptible” are phenotypical reactions to pathotype 3 of *P. brassicae*. The percentage indicates the rate of *Rcr1* identification in plants using the marker.

### Transcriptome profiling based on RNA-seq

Inoculated F_1_ seedlings from the cross ACDC × FN were bulked (R and S) based on MAS and examined for global transcriptomes using RNA-seq. Approximately 856 million raw reads were generated from a total of six pooled samples. About 92% of them passed the quality control standard, yielding 784 million of clean reads (Table 3). About 60% of the total reads were mapped to the *B. rapa* reference genome, with 97% of them being uniquely mapped, while 33% of the total reads were unmapped.

**Table 3:** **Summary of the RNA-sequencing reads from inoculated resistant and susceptible** ***B. rapa*** **root samples (F**_**1**_ **)**

Reads	Amount	Percentage of total raw reads
Total raw reads	856,009,740	100%
Average reads per sample	142,668,290	Not applicable
Total clean reads	783,978,544	92%
Total mapped reads	502,147,812	59%
Perfect match	218,073,408	25%
≤5 bp mismatch	284,074,404	33%
Unique match	485,652,620	57%
Multi-position match	16,495,192	2%
Total unmapped reads	281,830,732	33%

A total of 41,018 genes were annotated in the *B. rapa* reference genome sequence (v1.2). Transcripts of 36,221 of these genes were detected based on RPKM calculations (data not shown), and among them, more than 75% (27,322) had a coverage of 90% or higher by the mapped reads. A total of 2,212 differentially expressed genes (DEGs) were identified in this study (Additional file 1: Table S3), with 1,246 genes upregulated and 966 down-regulated in the R samples relative to S samples. Almost all genes in the fine mapped *Rcr1* region between 24.32 Mb and 24.35 Mb of A03 were expressed, but only a few of them were identified as DEGs and most of them showed no difference in expression levels between inoculated R and S. Interestingly, two of the TIR-NBS-LRR genes (*Bra019412*, *Bra019413*) within this region were significantly upregulated in the inoculated R treatment relative to the inoculated S treatment.

**Figure 5:**
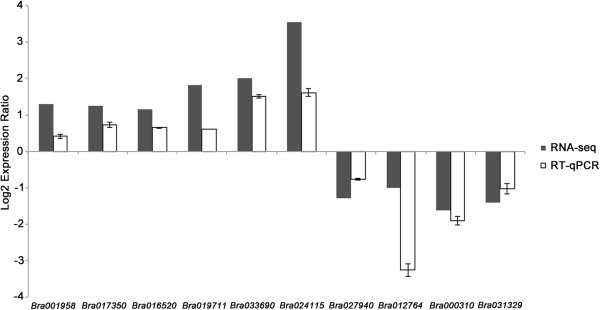
**Validation of RPKM-calculated expression ratios for selected differentially expressed genes (DEGs) using RT-qPCR.** RPKM values from RNA-seq are denoted in black, and RT-qPCR results in while. Capped lines represent the standard deviations from three biological replicates.

RT-qPCR analysis of 10 selected genes over the same R and S bulk samples showed a trend consistent with that of RPKM calculations and statistical analyses of transcript data (Figure 5). The RT-qPCR data confirmed up-regulation of a class-1 non-symbiotic hemoglobin (*Bra001958*), erd12 protein (*Bra017350*), protein tify 10a (*Bra016520*), s-adenosyl-l-methionine:carboxyl methyltransferase family protein (*Bra019711*), transcriptional factor bhlb92-like protein (*Bra033690*) and transcriptional factor bhlh35 (*Bra024115*) genes in resistant samples detected via RNA-seq. The data also verified down-regulation of a chitinase-like protein (*Bra027940*), ralf-like 33 protein (*Bra012764*), endochitinase isolog (*Bra00031*0) and cell-wall-protein-like protein (*Bra031329*) genes (Figure 5) as indicated in RNA-seq analysis.

### Annotation of DEGs

**Figure 6:**
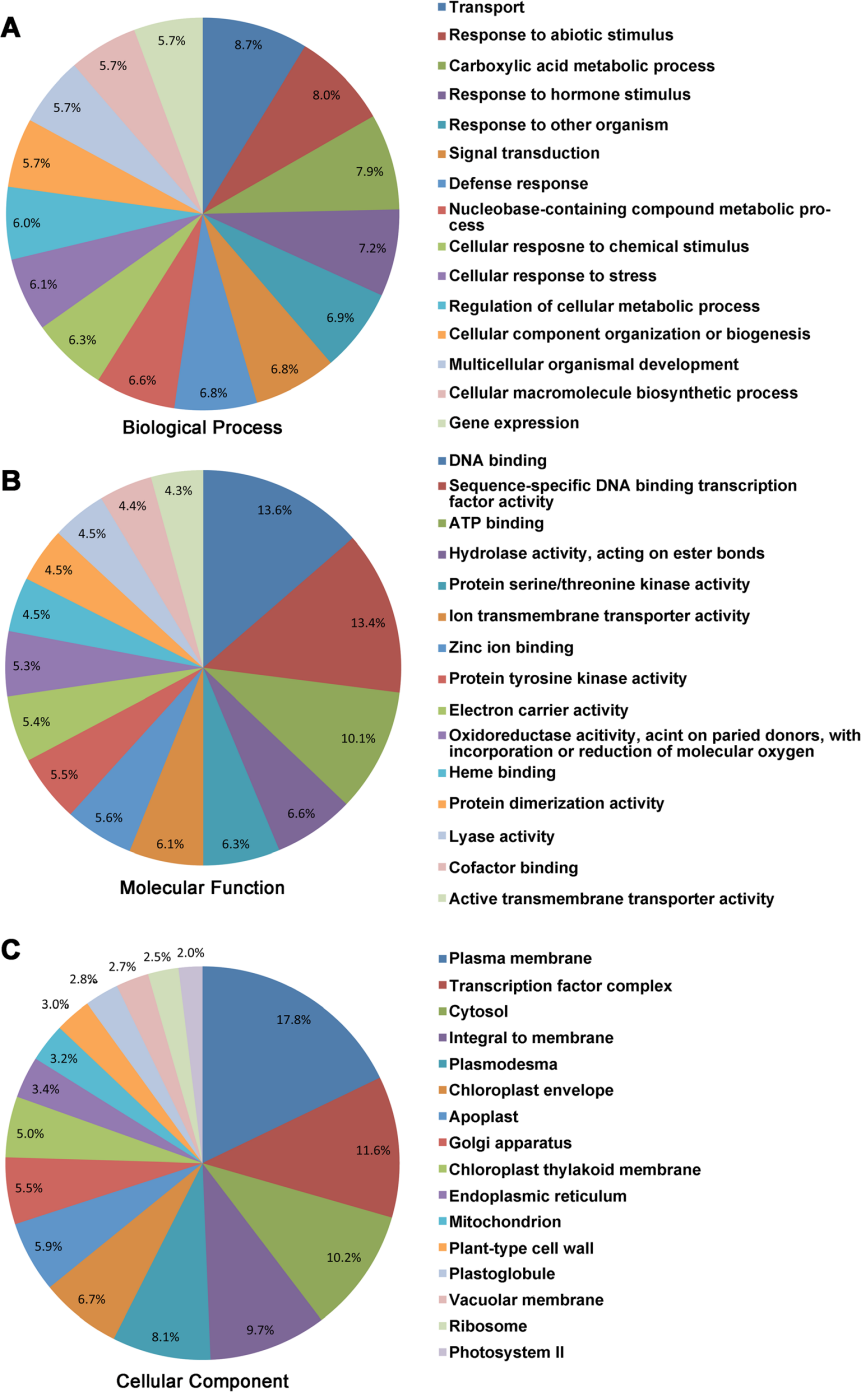
**GO terms associated with upregulated DEGs. A) GO terms in the Biological Process category. B)** GO terms in the Molecular Functions. **C)** GO terms in Cellular Components. The values labeled in the pie charts of panel A, B and C are the percentage of DEGs annotated with the corresponding GO term relative to the total DEGs.

**Figure 7:**
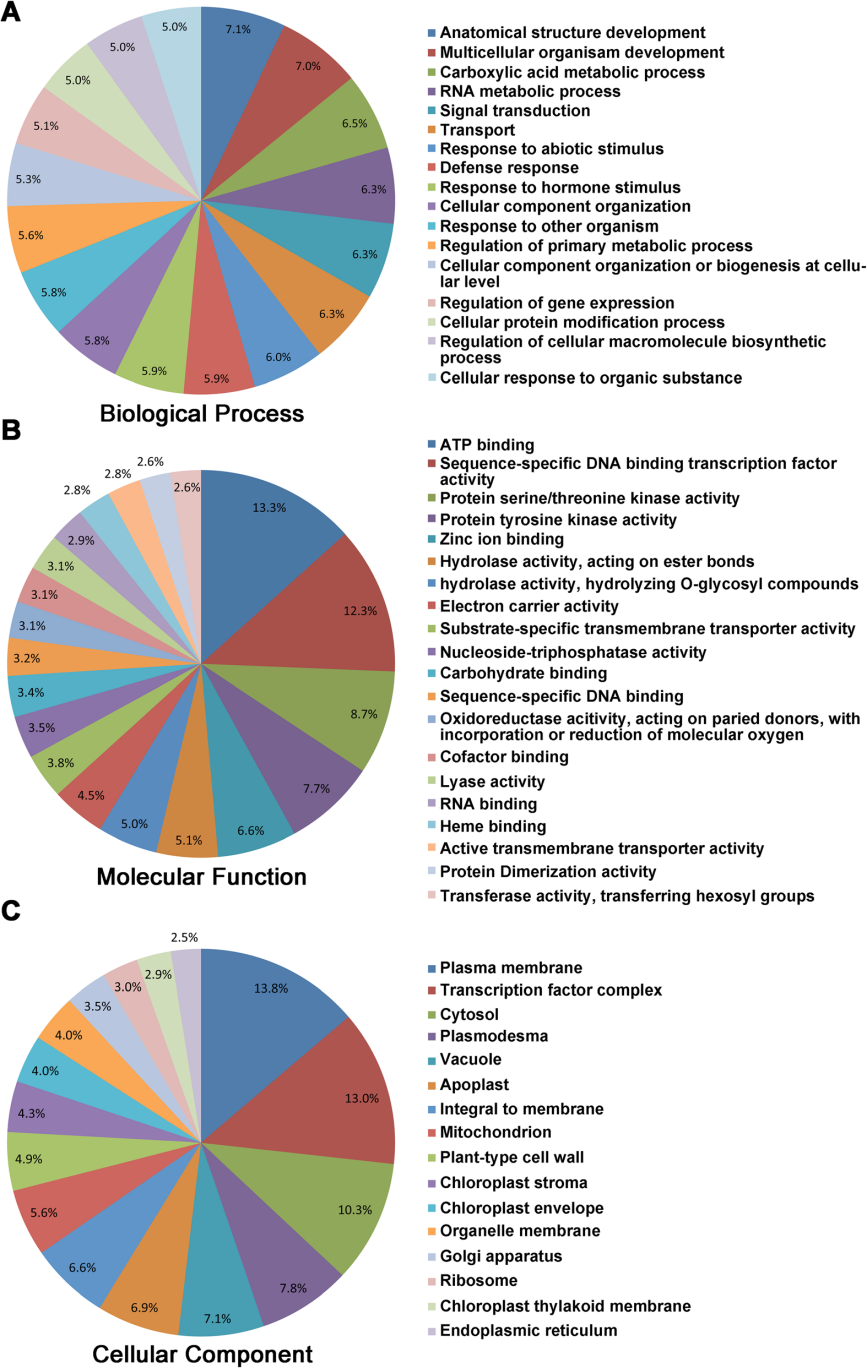
**GO terms associated with down-regulated DEGs. A)** GO terms in the Biological Process category. **B)** GO terms in Molecular Functions. **C)** GO terms in Cellular Component. The values labeled in the pie charts of panel A, B and C are the percentage of DEGs annotated with the corresponding GO term relative to the total DEGs.

The DEGs were functionally annotated based on GO terms (Additional file 1: Table S4) and sorted into the GO-term biological process, molecular function and cellular component (Figure 6; Figure 7; Additional file 1: Table S4) using Blast2Go [39]. The statistics for GO-term mapping were provided in Additional file 1: Figure S1. A total of 55 DEGs retrieved no hits with BLAST (Additional file 1: Table S4).

The annotated DEGs with upregulated patterns in R samples fell mainly into 15 categories of biological process (Figure 6A). The GO term “defense response” (6.8%) was also one of the major categories identified. Other upregulated biological processes of GO terms included signal transduction, various metabolic/biosynthetic processes and regulation of metabolic processes.

For molecular functions, several cellular-component GO terms were identified, especially those associated with the plasma membrane representing the largest group (Figure 6C).

The GO terms for biological processes associated with down-regulated DEGs were mostly in the category of “anatomical-structural and multicellular-organismal development” (Figure 7A) and “regulation of primary metabolic process”. Most of the molecular functional GO terms associated with down-regulated DEGs were in the same categories as those of upregulated DEGs, although several unique terms were identified, including sequence-specific DNA binding transcription factor activity, hydrolase activity on O-glycosyl compounds, substrate-specific trans-membrane transporter activity, and nucleoside-triphosphatase activity (Figure 7B). Similarly, the majority of cellular-component GO terms of down-regulated DEGs fell into categories similar to those of upregulated DEGs, with only three new GO terms observed: vacuole, chloroplast stromal and organelle membrane (Figure 7C).

Transcription factors (TF) were also characterized broadly for DEGs; a total of 92 upregulated and 57 down-regulated DEGs were grouped into seven types of TF, based on their conserved structures. For upregulated DEGs, 18 of them belong to WRKY, 15 are MYB domain-containing TFs, 10 are ethylene (ET)-responsive TFs, 15 are *bhlh*-domain containing TFs, 13 belong to the AP2/ERF family, 6 are heat-stress related TFs, and the remaining 15 belong to “other” TF families (Table 4). For down-regulated DEGs, 8, 6, 5, 2, 8, 0 and 28 of them fell into the respective TF families (Table 5).

**Table 4:** **Up-regulated differentially expressed genes (DEGs) annotated as transcription factors (TFs)**

TF annotations	Log2-fold change	Gene ID
WRKY domain containing	1.0 ~ 7.8	*Bra008454, Bra014693, Bra013708, Bra000202, Bra009734, Bra016975, Bra005104, Bra003588, Bra019123, Bra020814, Bra023983, Bra011299, Bra008435, Bra020628, Bra016535, Bra026467, Bra040926, Bra013584*
MYB domain containing	1.1 ~ 4.4	*Bra025681, Bra006977, Bra029349, Bra037837, Bra039067, Bra027389, Bra030812, Bra040274, Bra029553, Bra008539, Bra015939, Bra029582, Bra013000, Bra008131, Bra001202*
Ethylene-responsive	1.0 ~ 3.0	*Bra031903, Bra017235, Bra028703, Bra034249, Bra012345, Bra029302, Bra026280, Bra023748, Bra028291, Bra017656*
*Bhlh* domain containing	1.1 ~ 3.5	*Bra024115, Bra011152, Bra027501, Bra033690, Bra000291, Bra036640, Bra039926, Bra035639, Bra011790, Bra001168, Bra010467, Bra037887, Bra018461, Bra004532, Bra030208*
AP2/ERF family	1.0 ~ 3.8	*Bra037794, Bra029147, Bra035919, Bra019087, Bra027612, Bra007975, Bra028009, Bra016518, Bra017879, Bra027002, Bra032665, Bra030255, Bra011002*
Heat stress	1.0 ~ 2.2	*Bra000557, Bra000235, Bra012829, Bra007739, Bra008593, Bra012828*
Other	1.1 ~ 7.6	*Bra036071, Bra001648, Bra022189 Bra031691, Bra005688, Bra019154, Bra008113, Bra012500, Bra025398, Bra001290, Bra036483, Bra016389, Bra012887, Bra007869, Bra015582*

**Table 5:** **Down-regulated differentially expressed genes (DEGs) annotated as transcription factors (TFs)**

TF annotations	Log2-fold change	Gene ID
WRKY domain containing	−1.0 ~ −2.3	*Bra027480, Bra004864, Bra020546, Bra031900, Bra030273, Bra006178, Bra032340, Bra030178*
MYB domain containing	−1.0 ~ −4.6	*Bra002107, Bra033291, Bra036412, Bra036202, Bra001311, Bra038774*
Ethylene-responsive	~ − 1.3	*Bra036360, Bra002168*
Bhlh domain containing	−1.1 ~ −2.0	*Bra031852, Bra017024, Bra040856, Bra007228, Bra008716*
AP2/ERF family	−1.0 ~ −3.5	*Bra011782, Bra015478, Bra026949, Bra004878, Bra036536, Bra009824, Bra028690, Bra008460*
Heat stress	n/a	n/a
Other		*Bra010225, Bra010287, Bra003483, Bra014478, Bra005777, Bra032727, Bra011190, Bra018027, Bra002595, Bra002004, Bra005396, Bra030783, Bra036854, Bra000301, Bra015960, Bra028824, Bra007727, Bra039127, Bra010875, Bra029778, Bra035077, Bra012583, Bra014657, Bra001032, Bra022968, Bra022225, Bra031302, Bra014971*

### Biological-process GO terms for up- and down-regulated DEGs

**Figure 8:**
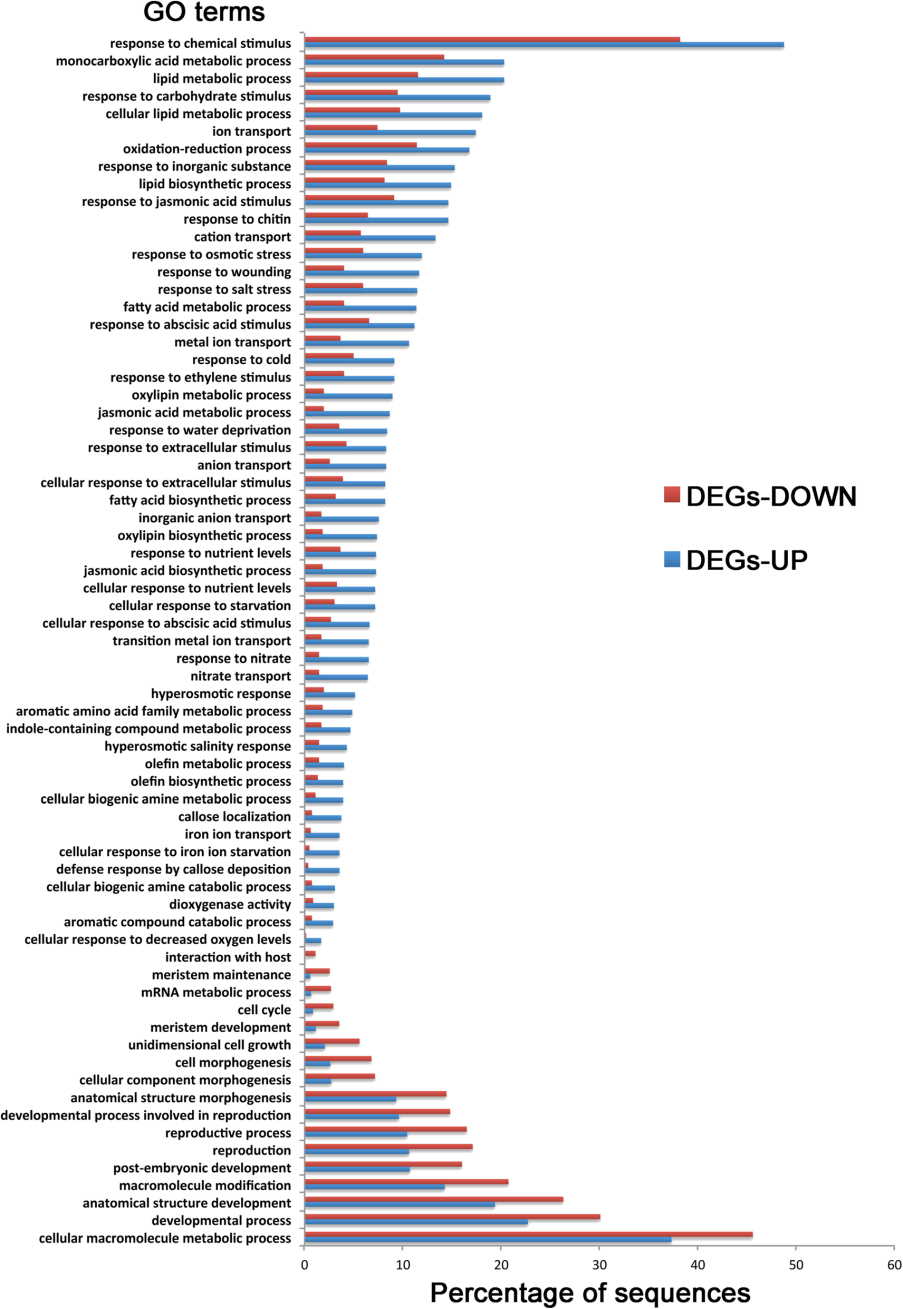
**Comparison of GO annotations (in Biological Process) of up- and down-regulated DEGs.** Blue bars represent the upregulated DEGs while red bars represent down-regulated DEGs. The values were the percentage of DEGs annotated with the corresponding GO terms relative to the total up- or down-regulated DEGs. Statistics of enrichment analysis are presented in the Additional file 1: Table S5.

Analysis using the Fisher’s Exact Test in Blast2GO identified the enrichment associated with up- and down-regulated DEGs; a total of 89 biological-process GO terms displayed significant enrichment (Figure 8), with 72 of them associated with upregulated and 17 with down-regulated DEGs. Most of these enriched GO terms were related to “responses”, including those to chemical and hormone stimuli. The results were similar for metabolic-process GO terms, with most of the enriched term being related to “responses”. Among 72 enriched GO terms for upregulated DEGs, 7 were related to lipid metabolism, including lipid metabolic process, cellular lipid metabolism, lipid biosynthesis, fatty acid metabolism, fatty acid biosynthesis, oxylipin metabolism and biosynthesis. Several GO terms related to defense-related phytohormones, including jasmonic acid and ethylene, but not salicylic acid (SA), were also highly enriched in inoculated R plants relative to those in inoculated S plants (Figure 8). A total of 214 DEGs were annotated under GO terms with SA-related biological processes, including “response to SA stimulus”, “SA biosynthetic process “systemic acquired resistance, SA-mediated signaling pathway” etc. (Additional file 1: Table S3), but none of them was significantly enriched in either up- or down-regulated GO terms, as determined with the Fisher’s Exact Test. Noticeably, genes associated with several common defense responses were significantly upregulated, including “callose deposition”, “defense response by callose deposition” and “indole-containing compound metabolic process”. In contrast, most of the biological-process GO terms enriched in down-regulated DEGs were related to “development” and “morphogenesis”, including developmental process, anatomical structure development, anatomical structure morphogenesis and uni-dimensional cell growth (Figure 8).

### Transcript analysis of selected defence-related DEGs using RT-qPCR

**Figure 9:**
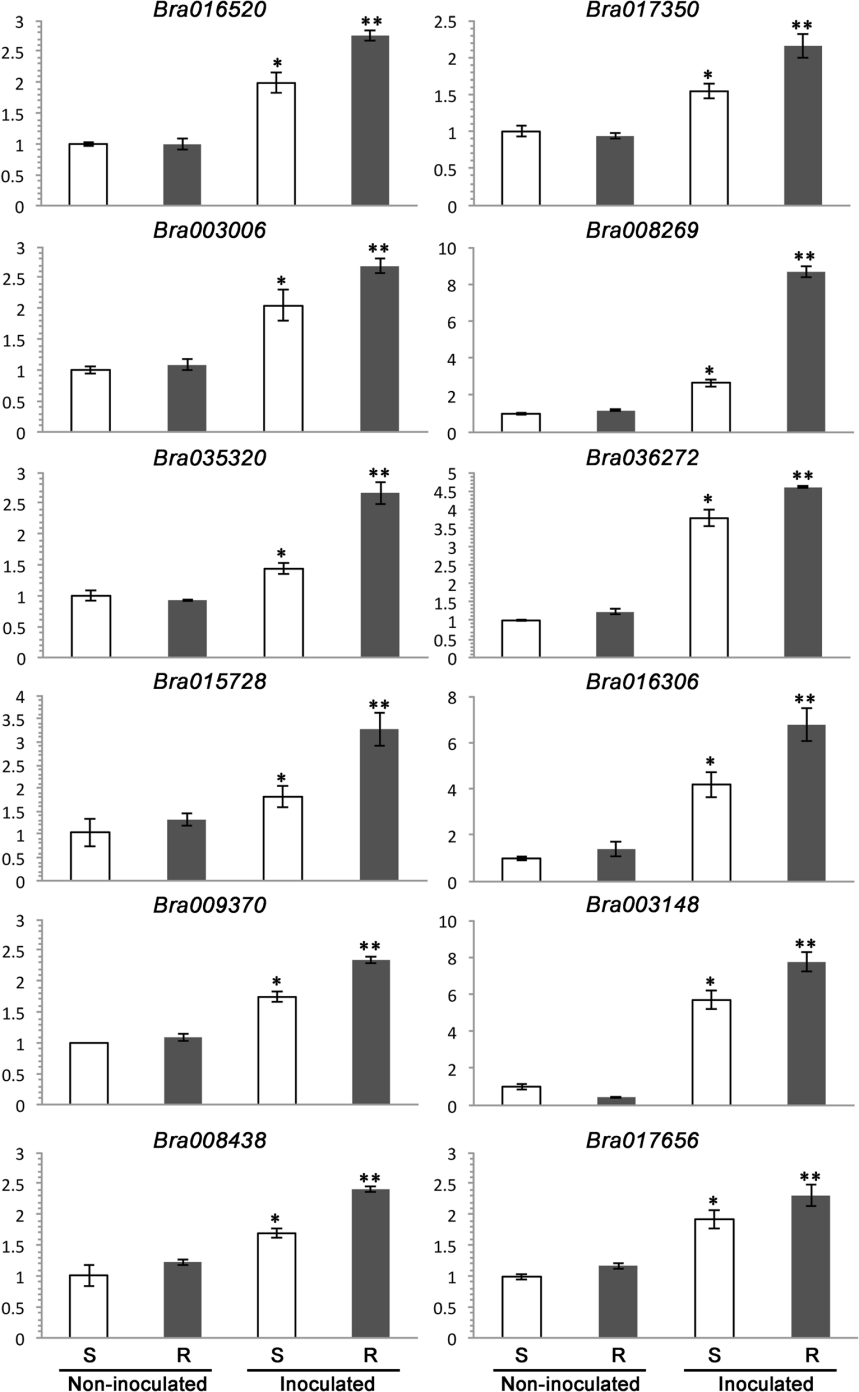
**The relative transcription quantity (RTQ) measured with RT-qPCR for selected upregulated DEGs identified in RNA-seq.** The treatments were S-susceptible (without *Rcr1*) and R-resistant (with *Rcr1*) plants inoculated with *Plasmodiophora brassicae* or water (non-inoculated). The vertical axis represents RTQ against an endogenous control (the actin gene *Bra037560*). Treatments with one asterisk showed significantly higher RTQ (LDS, *P* < 0.05) than those without asterisk, but lower RTQ than those with two asterisks.

**Figure 10:**
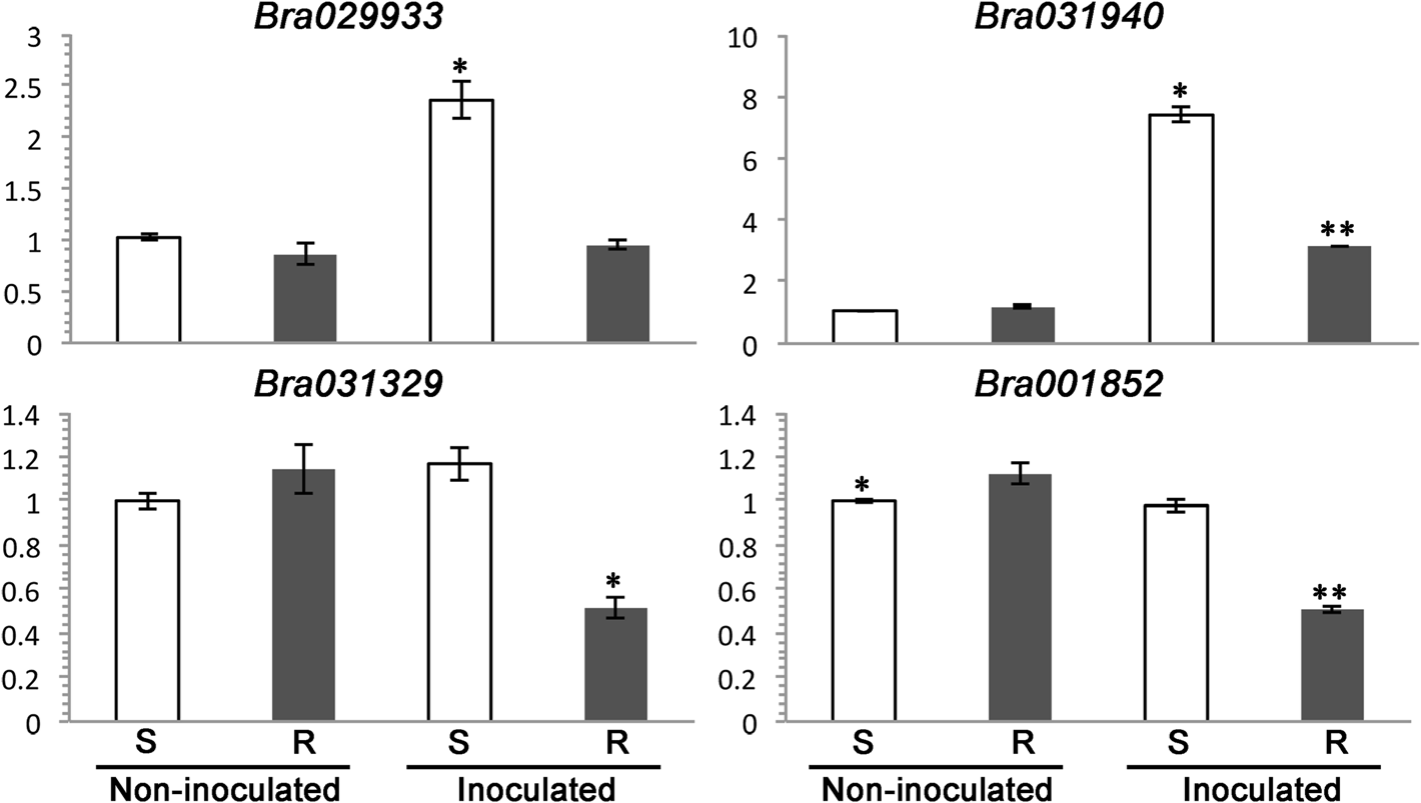
**The relative transcription quantity (RTQ) measured with RT-qPCR for selected down-regulated DEGs identified in RNA-seq.** The treatments were S-susceptible (without *Rcr1*) and R-resistant (with *Rcr1*) plants inoculated with *Plasmodiophora brassicae* or water (non-inoculated). The vertical axis represents RTQ against an endogenous control (the actin gene *Bra037560*). Treatments with one asterisk had a significantly different level of RTQ (LDS, *P* < 0.05) relative to without asterisk or with two asterisks.

Based on RNA-seq analysis, 12 strongly upregulated and 4 strongly down-regulated DEGs (based on RPKM -Reads Per Kilobase of transcriptome per million Mapped reads), involved possibly in resistance based on the functional annotation (Additional file 1: Table S4, with yellow highlight), were subjected to RT-qPCR analysis. All of the 12 upregulated DEGs displayed similar transcriptional patterns; the transcript levels, based on their relative quantity, were comparable for R and S plants without pathogen inoculation but significantly higher in R plants after inoculation (Figure 9). The 4 down-regulated DEGs, however, showed different transcriptional patterns; *Bra029933* and *Bra031940* displayed comparable transcript levels in non-inoculated S and R plants but these genes were significantly induced by *P. brassicae* in S plants and suppressed in R plants (Figure 10). *Bra031329* and *Bra001852* did not show significant induction by the pathogen in S plants relative to those in non-inoculated S or R plants, but suppressed in inoculated R plants (Figure 10). Two of the TIR-NBS-LRR genes (*Bra019412*, *Bra019413*) residing in the *Rcr1* region were also identified as DEGs in the RNA-seq, but were not included in the RT-qPCR test due to only moderate RPKM values. Preliminary RT-qPCR trials on DEGs with moderate RPKM showed unsatisfactory amplification (data not shown).

## Discussion

### Mapping of the *Rcr1*gene and development of genetic markers

The CR gene *Rcr1* (formerly *Rpb1*) was mapped previously to a genomic region in the linkage group A03 flanked by the markers sN8591 and sR6340I [26]. Additional markers were developed in the current study using the *B. rapa* genome information, with the markers A3-020 and MS7-9 being much closer to the CR gene than sR6340I. Several markers were highly accurate in detecting *Rcr1* in both *B. rapa* and *B. napus* plants, especially when two flanking markers were used together. These markers will be useful for MAS in resistance breeding. *Rcr1* is the only CR gene reported in pak choy (*B. rapa* ssp. *chinensis*), but the original source of the gene is not known. Most of the CR genes identified in Chinese cabbage (*B. rapa* ssp. *pekinesis*) originated from European turnip [40]. Four CR genes have been mapped previously to the linkage group A03, including *CRa*[18, 41], *CRb*[21], *CRk*[22] and *Crr3*[20, 42]. *CRa* has also been cloned recently [23]. Based on the relationship between common markers and the location of these CR loci, Diederichsen *et al.*[10] suggested that *CRa* and *Crr3* are identical, allelic or at least closely linked to *CRb* and *CRk*. Recent work also indicated that *CRb* is likely in the same position as *CRa*, and both genes conferred resistance to pathotypes 3 and 4 but not to pathotypes 1 and 2 of *P. brassicae*[43]. Based on the linkage distance, *Rcr1* is close to both *CRa* and *CRb*. However, the cv. FN which carries *Rcr1* is highly resistant to both pathotypes 2 and 3 [17], suggesting a different resistance spectrum from that of *CRa* or *CRb*.

The mechanisms for clubroot resistance are not well understood. Among the 67 genes annotated within the region defined by sN8591 and A3-020 (Figure 2C), four TIR-NBS-LRR genes can be located (http://brassicadb.org/brad/). The protein family containing NBS and LRR domains is the largest class of *R* genes cloned so far [44]. *CRa* and *Crr1* isolated from *B. rapa* also encode TIR-NBS-LRR proteins [23, 24]. Therefore, it is possible that *Rcr1* is one or a cluster of these TIR-NBS-LRR genes in the mapped region.

### Transcriptome analysis and GO annotation of DEGs

Transcriptome profiling can provide insights into the mechanisms of disease resistance. This approach has been used to characterize several molecular components associated with clubroot disease development, especially the role of cytokinins on *A. thaliana*[27] and *B. juncea*[45]. However, no comparative analysis of gene transcription between clubroot resistant and susceptible plants was available. In our study, transcripts of 36,621 genes were analyzed via RNA-seq and 2,212 DEGs were identified between inoculated R and S plants. This number of DEGs is comparable to that observed in a previous transcriptome analysis between rosette and folding leaves of Chinese cabbage [46]. Fifty DEGs retrieved no hit in a BLAST search (Additional file 1: Table S2), indicating a number of unknown genes expressed in inoculated R plants. The GO terms “defense response” (6.8%) and “plasma membrane” (17.8%) accounted for a substantial portion of upregulated DEGs. Some of these genes may be candidates for further studies of clubroot resistance, because the plasma membrane is where most plant-pathogen interaction occurs (reviewed by Day and Graham 2007) [47]. Additionally, a large proportion of upregulated DEGs were annotated with the GO term Biological Process involved in responses to external stimuli (Figure 6A) and many of these GO terms were also significantly enriched for upregulated DEGs (Figure 8). These results indicate that many cellular activities in inoculated R plants were significantly upregulated at 15 dpi when root infection and pathogen colonization were taking place [48–51]. It also appears that infection may have caused differential activation or deactivation of certain genes in the host that may affect clubroot development on resistant and/or susceptible plants.

The enrichment analysis identified GO terms for Biological Process associated with both upregulated and down-regulated DEGs. Several lipid compounds were implied to play a role in the inoculated R plants. Lipids have been shown to play critical roles in detecting infection and may also have a key role in regulating gene transcription [52]. During infection by *P. brassicae*, the R plants appeared to be able to mobilize lipid biosynthesis and metabolism, because several genes involved in oxylipin biosynthetic and metabolic processes, including *Bra016520, Bra003006* and *Bra008269*, were upregulated substantially (Figure 8). One group of the most intensively studied oxylipins is jasmonates, likely due to their involvement in multiple plant biological processes [53]. GO terms associated with “response to jasmonic acid (JA) stimulus”, “JA metabolic process” and “JA biosynthetic process” were significantly enriched within the group of upregulated DEGs (Figure 8). This possibly indicates that jasmonates have a role in clubroot resistance mediated by *Rcr1*. Prost *et al*. [54] employed *in vitro* growth inhibition assays to evaluate 43 natural oxylipins and found that 41 of them had inhibitory effects on a wide range of plant pathogens. Similarly, jasmonates appear to play a role in induced resistance to clubroot caused by biofungicides [33, 36]. Oxylipins may also be used by both host and pathogen as regulatory compounds during the host-pathogen interaction (reviewed by Tsitsigiannis and Keller; Christensen and Kolomiets) [55, 56]. Further work is needed to confirm the specific role(s) of oxylipins in clubroot resistance.

### Potential molecular mechanisms for clubroot resistance

Several clubroot-resistance mechanisms identified previously were supported in the current study. For example, genes involved in olefin (ET is the simplest form of olefin) metabolic and biosynthetic processes and in response to ET stimulus, were upregulated in plants carrying *Rcr1* (Figure 8). ET had previously been shown to restrict club development in *A. thaliana*[57] and to have a role in induced resistance mediated by biofungicides [33, 36]. This result supports the current opinion that JA and ET may act synergistically in plant defense [58]. Reinforcement of plant cell wall was also indicated based on the enrichment of GO terms for upregulated DEGs associated with callose localization and deposition (Figure 8). The role of callose has been well documented in resistance to penetration of plant cell wall by fungal pathogens [59]. In Arabidopsis, genes encoding the synthesis of β-1,3 glucan (callose) were suppressed in a compatible interaction between *P. brassicae* and susceptible ecotype Col-0 [60]. In the current study, the gene *Bra012684*, encoding an expansin-like protein (Additional file 1: Table S4), showed the highest increase in transcript among all 1,246 DEGs upregulated. This may indicate a structural/composition alteration to the cell wall, which could affect secondary infection in epidermal and cortical cells. Some of the above-mentioned defense-related DEGs were analyzed further with RT-qPCR using inoculated and non-inoculated plants to determine transcript levels as affected by *P. brassicae* (Figure 9). The results demonstrated that the inoculation increased the expression of these genes in both S and R plants. Inoculated R plants, however, showed significantly stronger expression of these genes relative to inoculated S plants. This result is consistent with that of RNA-seq. In addition, genes involved in synthesis of indole-containing compounds were also upregulated in R seedlings. The anti-microbial compounds derived from this metabolic process typically include tryptophan-derived metabolites [61] and flavonoids converted from aromatic-acid phenylalanine [62]. These indicate potential involvement of secondary metabolites in clubroot resistance. Although several common resistance mechanisms are identified with RNA-seq, further analysis is needed to assess the relative importance of each mechanism in clubroot resistance conferred by *Rcr1*.

As described above, *Rcr1* may encode a TIR-NBS-LRR protein. The best characterized defense signaling pathway related to this class of R proteins involves the biosynthesis of salicylic acid (SA) and pathogenesis-related (PR) proteins, accompanied typically by a hypersensitive reaction (HR) [63]. Additionally, SA biosynthesis is linked frequently to host resistance against biotrophic pathogens [64]. In the current study, however, GO terms related to SA biosynthesis, metabolism or signaling processes were not significantly up-/down-regulated based on the enrichment analysis. This lack of substantial change in transcription of genes involved in SA biosynthesis has also been observed with induced resistance against clubroot on canola [33, 36]. Previous reports have suggested HR as one of the possible mechanisms for clubroot resistance [65, 66], but only two upregulated DEGs, i.e., *Bra013123*, and *Bra036984*, were identified as PR genes based on GO annotation (Additional file 1: Table S4) in the current study and there is no experimental evidence to link any of them to SA- or HR-related defense responses. In a cytological observation, Deora *et al.* found no evidence of HR with clubroot resistant canola cultivars [49, 51]. It is possible that SA signaling pathways may not play a critical role in clubroot resistance.

Another mechanism for disease resistance is suppression of metabolic processes in the host that are required for pathogenesis. Previous research had demonstrated that the level of auxin (indole 3-acetic acid, IAA) increased in roots during secondary infection by *P. brassicae*, likely as the result of enhanced biosynthesis and conversion of host auxin precursors induced by the pathogen [27]. In the current study, *Bra019369,* which encodes a SAUR family of proteins, was among the most highly induced genes (960 fold, Additional file 1: Table S3) in R plants relative to S plants. SAUR proteins are closely linked to auxin biosynthesis and signaling [67]. For example, *SAUR39* had been found to be a negative regulator of auxin biosynthesis and transport in rice [68]. With clubroot, pathogen-induced auxin metabolism had been linked to pathogenesis in Arabidopsis [27]. Additionally, several genes encoding the auxin-responsive GH3 family of proteins were upregulated in R plants. The GH3 family has been linked with increased basal immunity via suppressing pathogen-induced auxin accumulation in rice [69, 70]. In addition to manipulating plant auxin homeostasis, over-expression of GH3.5 in an activation-tagged mutant of Arabidopsis displayed enhanced biosynthesis of camalexin, the major phytoalexin found with pathogen infection in Arabidopsis [71, 72]. Since the biosynthesis of camalexin and auxin were derived from the common precursor tryptophan [73], the metabolic stream may have been redirected to flow into the biosynthesis of phytoalexin from auxin in the R plants. Taken together, the results indicate that pathogen-activated auxin synthesis might have been suppressed or disrupted in R plants.

Most of the enriched GO terms associated with down-regulated DEGs were related to growth and development, including cell cycle, uni-dimensional cell growth, anatomical structure morphogenesis, and cell morphogenesis (Figure 7). Down-regulation of these genes may play a role in the resistance mediated by *Rcr1* because hypertrophy, the most typical symptom of clubroot [50], is related positively to these physiological activities. Expression of genes involved in cell enlargement and proliferation was inhibited in an Arabidopsis line partially resistant to *P. brassicae*, relative to the susceptible reaction [29]. In the current study, two of the genes (*Bra029933* and *Bra031940*) annotated for the GO term “uni-dimensional cell growth” were upregulated by the pathogen in inoculated S plants (Figure 10), as opposed to the same genes in inoculated R plants that changed little relative to those in non-inoculated plants. Down-regulation of these DEGs possibly works in conjunction with up-regulation of defense-related genes described above in resulting in a resistant outcome in plants carrying *Rcr1*. It is not clear if down-regulation of growth/development DEGs is directly related to the suppression of auxin-dependent metabolism via enhanced expression of *Bra019369* or GH3 genes observed. However, the evidence indicates that one or more processes related to auxin biosynthesis and cell growth/development are disrupted in R plants, with infection by *P. brassicae*.

RNA-seq analysis revealed that the clubroot resistance conferred by *Rcr1* involves complex mechanisms via a variety of biological processes controlled likely by corresponding transcription factors (TFs). As expected, 92 upregulated DEGs were identified based on their conserved structures as TFs responsible for activation of several biological processes potentially involved in disease resistance (Table 4). Consistent with the roles of ET and JA discussed above, ET-responsive TFs including the AP2/ERF-family TFs (subfamily of ET-response factors) [74] and *bhlh*-family TFs (regulating JA responses) [58] were upregulated to relatively high levels (2-14 fold). WRKY-family TFs were also upregulated. Their involvement in disease resistance, including effector-triggered immunity, has been suggested previously [75]. MYB-family proteins may be involved in secondary metabolism including flavonoid [76] and secondary cell-wall biosynthesis [77]. These biological functions may contribute to clubroot resistance by generating anti-microbial metabolites and strengthening host cell walls. Several heat-stress and “other” types of TFs were also identified, but their role in resistance is not understood. It is interesting that no heat-stress TFs were identified in the down-regulated DEGs, but the significance of this observation is unclear. Only a small number of defense-related DEGs were present in the 57 down-regulated DEGs sorted into the TF groups. The other down-regulated DEGs in TF groups are involved generally in cellular growth and development. In other words, these down-regulated TFs may represent targets that *P. brassicae* up-regulates during pathogenesis in S plants.

## Conclusions

Genetic resistance is the cornerstone for management of clubroot on canola. In this study, we characterized the CR gene *Rcr1*, based on genetic mapping and transcript analysis, to develop markers for MAS and decipher molecular mechanisms of resistance associated with *Rcr1*. RNA-seq analysis identified a range of biological processes potentially involved in clubroot resistance, consisting of both up-regulated defense-related and suppressed pathogenesis-related responses. This information is highly useful to design a breeding strategy based on modes of action of CR genes to achieve strong and durable clubroot resistance. Although SA biosynthesis is often linked to plant resistance against biotrophic pathogens, genes involved in SA biosynthetic pathways were not activated in inoculated plants carrying *Rcr1*. In contrast, genes involved in JA/ET and callose biosynthesis were upregulated substantially. The biosynthesis or signaling of JA/ET has not been identified previously for resistance to clubroot. Further research is needed to confirm specific roles of these phytohormones in clubroot resistance mediated by *Rcr1*. It will also be useful to look at these phytohormones in association with other CR genes.

## Methods

### Plant materials, pathogen inoculum, and inoculation

The hybrid pak choy cv. FN (Evergreen Y.H. Enterprises, Anaheim, CA), highly resistant to each of the five pathotypes of *P. brassicae* found in Canada [17], was used to pollinate the doubled haploid (DH) canola line ACDC (*B. rapa*) developed at AAFC Saskatoon Research Centre. This DH line is self-compatible and highly susceptible to pathotype 3 of *P. brassicae*, a dominant pathotype on canola in western Canada [78]. Seeds were sown in Sunshine #3 soil-less planting mix (SunGro Horticulture, Vancouver, BC) in tall plastic pots called “conetainers” (5-cm diam, 20-cm tall, Steuwe & Sons, Corvalis, OR), and plants were transplanted later into the same growth medium in 15-cm-diam. pots (1 plant/pot) at 5 weeks after seeding. The planting mix was amended with 1% (w/v) 16-8-12 (N:P:K) control-released fertilizer. Plants were kept in a greenhouse (22/18°C, day/night) with a 14-h photoperiod (230 μmol/m^2^/s at the canopy level) or in a growth room at 23/20°C and 14-h photoperiod (512 μmol/m^2^/s).

A field population of *P. brassicae* (Leduc-AB-2010)*,* consisting primarily of pathotype 3 of *P. brassicae*, was used for inoculation throughout the study. Mature clubroot galls filled with pathogen resting spores were dried at room temperature for 2 weeks and stored at −20°C until use. The inoculum was prepared as a resting-spore suspension using the method described by [33], with the concentration adjusted to 1 × 10^7^spores/mL. For inoculation, 5 mL of a resting-spore suspension were pipetted around the seed in each conetainer immediately after sowing to result in an inoculum dose of about 1 × 10^6^ spores/g growth medium. Inoculated conetainers were kept in the growth room and watered daily for 2 weeks to maintain a high level of soil moisture to facilitate infection. ACDC was used as a susceptible control in all inoculated trials. Non-inoculated plants would not develop any visible clubroot symptoms [79].

Reciprocal crosses were made between the hybrid cv. FN and ACDC to produce F_1_ progenies. Five well developed buds per female plant were kept for crossing, and the other flowers and small buds were removed. Each bud that remained was opened and the anthers removed carefully with a pair of forceps. Anthers were collected from newly opened flowers of donor plants, and pollen grains were dusted to pistils of the female plants with a small paintbrush. Each pollenated plant was covered with a plastic crossing bag for 5 days..

### The bioassay for clubroot test

The parents and their progenies were inoculated as described above, and plants were assessed at 5 weeks after seeding for clubroot severity using a 0–3 scale [80]. A rating of 0 was considered resistant (R) and 1-3 susceptible (S). Each FN plant used in the crosses was resistant to clubroot. Due to heterozygosity of cv. FN, F_1_ populations resulting from the reciprocal crosses between ACDC and FN segregated for resistance and susceptibility. The goodness of fit for the segregation was analyzed using the Chi-square (*X*^*2*^) Test [81]. For fine mapping and RNA-seq, only the F_1_ population from the ACDC (female) × FN (male) cross was used.

### Fine mapping based on marker analysis

Simple sequence repeat (SSR) markers (http://aafc-aac.usask.ca/BrassicaMAST/) were used for the fine mapping work. Over 2,000 SSR markers had been developed at AAFC Saskatoon Research Centre, which distributed on 19 linkage groups of *B. napus*. A total of 97 polymorphic SSR markers on the A genome were identified and *Rcr1* was rough mapped to A03 [26]. SSR markers flanking *Rcr1* were further used to screen a segregating F_1_ (testcross) population of 1,587 plants, each of which was also tested for clubroot reaction.

The MegaBACE 1000 DNA Analyser (GE Healthcare, Mississauga, CA), a capillary-array electrophoresis system with automated gel matrix replacement, sample injection, DNA separation and base calling, was used for SSR marker analysis. PCR products were amplified with selected polymorphic markers, and forward primers labeled by adding fluorescent phosphoramidite (PE Biosystems, Foster City, CA) as HEX (yellow), TET (green) or 6-FAM (blue), and segregated on the MegaBACE. Laser excitation and confocal laser scanning are used to excite and detect fluorescent dye-labelled DNA fragments, respectively, as they migrate past a detection window. The fragment analysis was carried out using the Genetic Fragment Profiler Software Suit V1.2 (GE Healthcare). DNA fragments from the F_1_ population could be separated into three bands (1, 2 and 3) by some markers; the band 1 and 2 were from the heterozygous FN, and band 3 from homozygous ACDC. Since we were more interested in the R allele (band 1), only two genotypes were grouped based on marker analysis; genotypes with the band 1 and 3 were scored as “h” and those with band 3 and 2 as “a”. The linkage analysis was performed using JoinMap 4.1 [82]. DNA sequences identified within the region of the CR gene flanked by SSR markers were used to search for similar *B. rapa* genomic DNA sequences at http://brassicadb.org/brad/, and the information used to develop CAPS markers. PCR primers were designed using Primer3 (http://frodo.wi.mit.edu/). Protocols described previously [83] were followed for amplification reactions and cleavage.

DNA sample preparation and PCR conditions: DNA was extracted following the method described previously [84] with these slight modifications: Freeze-dried leaf samples were incubated with extraction buffer (2% CTAB; pH 8.0) at 65°C, followed by chloroform-isoamylalcohol (24:1, v/v) extraction and alcohol precipitation. RNA was eliminated by adding 1/10 volume of 10 mg/mL RNase A. The DNA concentration was estimated using the NanoDrop ND-2000c (Thermo Scientific, Wilmington, DE) and adjusted to10 ng/μL with sterile Milli-Q water. A PCR mixture containing 0.5 μL each of forward and reverse primers (5 μmol/L), 4 μL 10 ng/μL genomic DNA, 5 μL AmpliTaq Master Mix (Life technologies, Burlington, CA) was pipetted to a 384-well PCR plate. The reaction loosely linked to these CR genes conditions were as follows: denaturation at 95°C for 10 min, followed by 8 cycles of 94°C for 15 s, 50°C for 15 s, and 72°C for 30 s; then 27 cycles of 89°C for 15 s, 50°C for 15 s, 72°C for 30 s, and a final extension at 72°C for 10 min.

### Validation of selected markers for detection of *Rcr1*in backcross populations

Four markers, i.e., sN8591, sR6340I, sB4889B and sS2093, were examined to confirm the presence of *Rcr1* in resistance BC_1_ (BC_1_F_1_) progeny derived from backcrossing the canola DH lines BH11-17938 (*B. rapa*) and SV11-17667 (*B. napus*), respectively, with cv. FN. These two populations were different from that used for mapping and RNA-seq (ACDC × cv. FN); they were produced during introgression of *Rcr1* into AAFC canola breeding lines. The purpose of this experiment was to assess the selected markers for the presence of *Rcr1* during resistance introgression. To produce BC_1_ progenies, about 20 plants were produced for each donor F_1_ population (*B. rapa*, *B. napus*), with five resistant plants selected based on their clubroot reaction, assessed as described previously. The crosses of recurrent breeding lines (female) × resistant F_1_ (male) lines were made and BC_1_ seeds were bulked in case some of the “resistance plants” were misidentified due to escape. A population of 176 plants were tested from each of the *B. rapa* and *B. napus* BC_1_ populations. The clubroot reaction of each plant was assessed as described previously. Marker detection for *Rcr1* was performed on the MegaBACE and compared with phenotype data for each plant.

### RNA isolation, RNA-seq and data analysis

The F_1_ population of ACDA × cv. FN used for fine mapping was also used for RNA-seq. The whole root system was cut from each plant at 15 days post inoculation (dpi). At this point, infection of the root cortex has been initiated but clubbing symptoms are not yet visible in susceptible plants [48, 49]. The roots were dug out, rinsed with tap water, and separated into R and S groups using the flanking markers MS1-3 (5′-AAAACAAATATCCACCACG-3′ and 5′-CTCAATCCCACAAACCTG-3′) and A3-028 (5′-GAGGCCTCCTTTTCTGGTTT-3′ and 5′-CCGGAGAAGTTTGATTCGAG-3′). These markers were located at 24.06 Mb and 25.36 Mb, respectively, in the linkage group A03. The effectiveness of these markers in detecting *Rcr1* was verified prior to the experiment using the F_1_ population consisting of 50 plants, and the result matched 100% with that of clubroot reaction assessed at 5 weeks after inoculation (data not shown)_._ To verify root infection by 15 dpi, 10 plants from each of the inoculated R and S groups (based on marker detection) were kept in pots until 21 dpi for examination of root symptoms. All of the S plants showed tiny galls, indicating that root infection had likely occurred at 15 dpi. All of the R plants were free of galls (data not shown). There were four treatments, consisting of inoculated and non-inoculated R and S plants. The entire root system of nine random plants were bulked to produce a biological replicate, with three replicates per treatment (R or S, inoculated and non-inoculated). This bulk sampling method has been used previously in studies for marker identification and transcriptome analysis [85, 86]. The total RNA from each replicate (9 roots, bulked) was isolated using an RNeasy Plant Mini Kit (Qiagen; Toronto, CA) with on-column deoxyribonuclease (DNase) digestion using a Qiagen RNase-Free DNase Set following the manufacturer’s instruction. The RNA concentration and quality were checked using Nanodrop 2000c and Agilent Bioanalyzer 2100 (Agilent Technologies; Mississauga, CA) respectively, to ensure that the RNA integrity number (RIN) was greater than 9 for each sample.

RNA-seq was carried out on each inoculated S and R sample using the Illumina Hiseq 2500 platform at Plant Biotechnology Institute-National Research Council (NRC-PBI, Saskatoon, Canada). The cDNA library was prepared using TruSeq RNA Sample Preparation Kits v2 (Illumina; San Diego, CA). The raw reads were filtered to remove sequencing adapters, as well as low-quality reads (>5% unknown bases, or >50% of the bases with a quality <5), to generate “clean” reads that subsequently were aligned to the Chinese cabbage (*B. rapa* ssp. *pekinesis*) Chiifu genome (V1.2; http://brassicadb.org/brad) using the SOA Paligner/SOAP2 package [87] with ≤ 2 mismatches.

Gene expression was calculated using the RPKM method, because comparison analysis [88] showed a better correlation between RPKM and qPCR than between FPKM (Fragments Per Kilobase of exon per Million fragments mapped) and qPCR. Identification of DEGs followed the protocol developed by [89], and a log2-based ratio was calculated to indicate fold changes in gene expression levels between R and S samples based on the results from the three biological replicates. The false discovery rate (FDR) was used to measure the threshold of the *P*-value for the three tests, and a threshold FDR ≤ 0.001 and the absolute value of log2 ratio ≥ 1 were used to identify DEGs [90].

### Annotation of differentially expressed genes (DEGs)

The DEGs for R samples were separated initially into the DEGs-UP and DEGs-DOWN groups relative to the gene expression observed in S samples. Both groups were subjected to annotation of gene ontology (GO) using Blast2GO [39] to run BLASTX algorithms against the non-redundant protein database from the National Center for Biotechnology Information (http://www.ncbi.nlm.nih.gov). All BLAST hits were mapped to the functional information stored in the GO database to retrieve GO terms associated with the hits in the BLAST search. A GO-term pool generated by GO mapping was used to annotate each of the sequences, with combined graphs generated and presented in the Results. The statistical assessment of annotation for DEGs-UP and DEGs-DOWN was performed using the Gossip package [91] integrated in Blast2GO.

### Real-time reverse transcription (RT) quantitative PCR (qPCR)

There were two purposes for conducting this procedure: 1) to provide a snap shot for the reliability of RNA-seq data; the Log_2_ fold of RPKM values for 10 highly activated or suppressed DEGs in RNA-seq were compared with the expression ratio (also on the Log_2_ scale) of the same set of genes in RT-qPCR; 2) to assess potential induction of defense-related genes with *P. brassicae* inoculation; transcription of 16 selected defense-related genes (12 upregulated and 4 down-regulated in RNA-seq) in inoculated and non-inoculated roots was quantified. The experiment was performed on the StepOne® Plus system (Life technologies). RNA samples from both inoculated and non-inoculated plants were prepared as described above. The primers (Additional file 1: Table S1) were designed using the Applied Biosystems Primer Express V3.0 (Life Technologies) and synthesized by Integrated DNA Technologies Inc. (Coralville, IA). Complementary DNA was synthesized using the Invitrogen SuperScirpt III First-strand Synthesis system (Life Technologies) from 1 μg of total RNA. PCR was conducted using the Power SYBR green master mix (Life technologies) following manufacturer’s instruction. Cycling conditions were 95°C for initial 10 min followed by 40 cycles of 15 s at 95°C, 30 s at 50°C and finally 30 s at 60°C. Melt-curve profiling and agarose gel electrophoresis were conducted to evaluate the specificity of the reaction and absence of primer dimers. The actin gene *Bra037560* was used as an endogenous control to normalize the expression level of target genes because of its consistent level of expression among the samples tested. The absolute expression levels for this reference gene, measured as RPKM in RNA-seq, were 601.6117144, 712.4030486 and 612.0688501 for three R replicates, and 593.0082633, 579.5445779, and 623.4046585 for three S replicates. The relative expression data were analyzed using the StepOne® software V2.2.2 (Life technologies). Three technical replicates were used for each cDNA sample and there were three samples (biological replicates) for each treatment. The log_2_-fold change observed with RT-qPCR was compared with the RNA-seq data. Analysis of variance and Fisher’s Least Significant Difference (*P* < 0.05) were performed using the software Statistical Product and Service Solutions (V20.0; IBM, Markham, CA ) to compare the relative transcription quantity for genes examined with RT-qPCR.

## Electronic supplementary material

Additional file 1: Figure S1: Statistics of GO term mapping by Blast2GO. **Table S1.** Sequences of the primers used for qPCR validation of selected gene expression. **Table S2.** GO annotations of genes residing in the fine mapped region. **Table S3.** Summary of identified DEGs. **Table S4.** Annotation of identified DEGs using Blast2GO. **Table S5.** Statistics of enrichment analysis for data presented in Figure 8. (ZIP 984 KB)

## Abbreviations

CR: Clubroot resistance
DEGs: Differentially expressed genes
FN: cv. Flower Nabana of *Brassica rapa* ssp. *chinensis*
R: Resistant
S: Susceptible
MS: Moderately susceptible
MAS: Marker-assisted selection
GO: Gene ontology
TF: Transcription factors
ET: Ethylene
JA: Jasmonic acid
SA: Salicylic acid
RPKM: Reads Per Kilobase of transcriptome per million reads Mapped
FPKM: Fragments Per Kilobase of exon per million fragments Mapped)
FDR: False discovery rate.
