# Supplementary figures and images for: Fine mapping of Rcr1 and analyses of its effect on transcriptome patterns during infection by Plasmodiophora brassicae
Source: BMC Genomics. 2014 Dec 23;15(1):1166. doi: 10.1186/1471-2164-15-1166 (PMC4326500; doi:10.1186/1471-2164-15-1166)

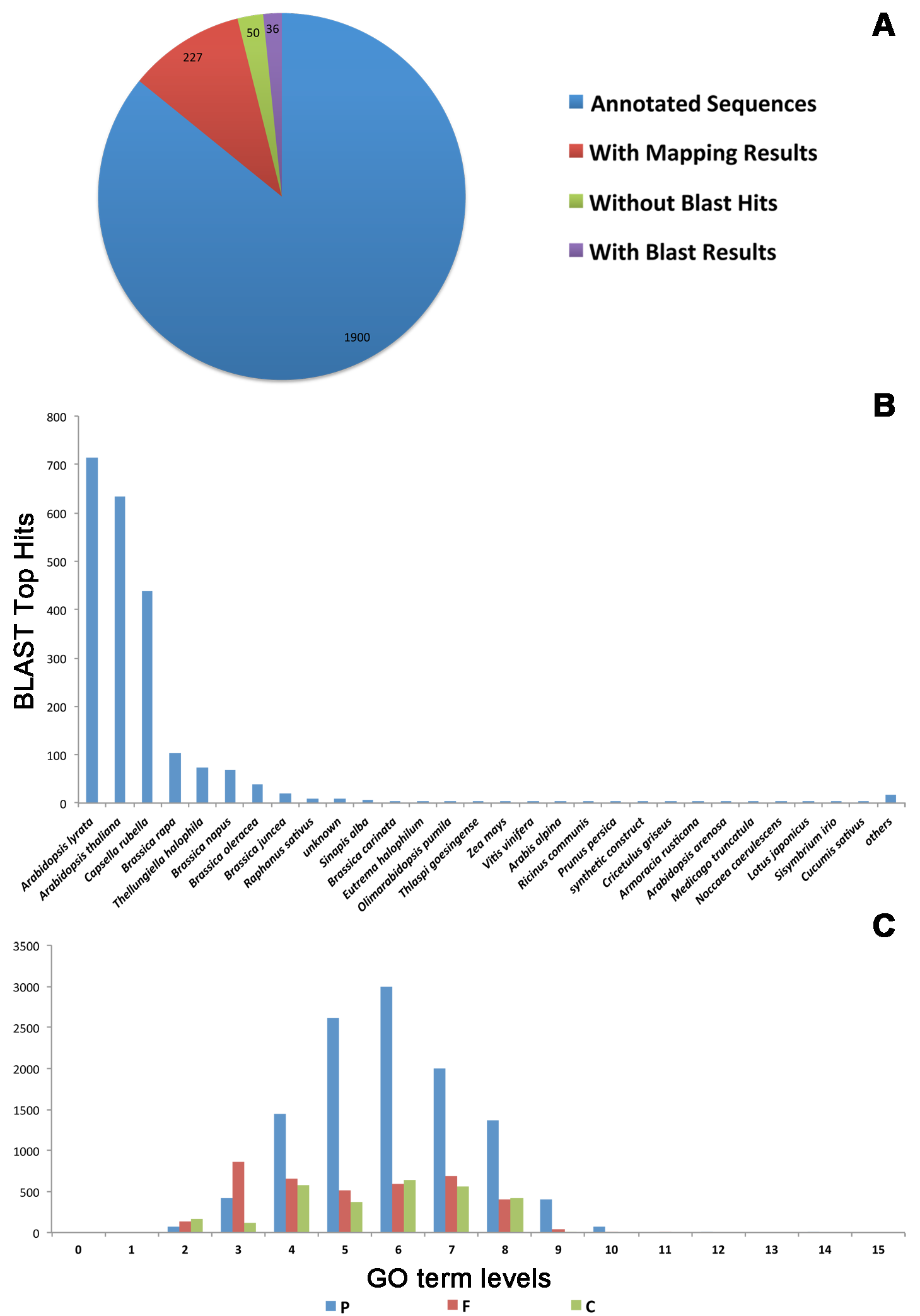

Supplement: Supplementary file 1 — Additional file 1: Figure S1: Statistics of GO term mapping by Blast2GO. Table S1. Sequences of the primers used for qPCR validation of selected gene expression. Table S2. GO annotations of genes residing in the fine mapped region. Table S3. Summary of identified DEGs. Table S4. Annotation of identified DEGs using Blast2GO. Table S5. Statistics of enrichment analysis for data presented in Figure 8. (ZIP 984 KB) [file 12864_2014_6989_MOESM1_ESM.zip › Figure S1 Statistics of GO term annotation_v2.tif]
